# Non-Destructive Direct Pericarp Thickness Measurement of Sorghum Kernels Using Extended-Focus Optical Coherence Microscopy

**DOI:** 10.3390/s23020707

**Published:** 2023-01-08

**Authors:** Dipankar Sen, Alma Fernández, Daniel Crozier, Brian Henrich, Alexei V. Sokolov, Marlan O. Scully, William L. Rooney, Aart J. Verhoef

**Affiliations:** 1 Department of Physics & Astronomy, Texas A&M University, TAMU 4242, College Station, TX 77843, USA; 2 Institute for Quantum Science & Engineering, Texas A&M University, TAMU 4242, College Station, TX 77843, USA; 3 Department of Soil and Crop Sciences, Texas A&M University, TAMU 2474, College Station, TX 77843, USA

**Keywords:** Fourier domain optical coherence microscopy, higher-order-mode fiber, long-period grating, Bessel beam, spatial resolution, sorghum, pericarp, phenotyping

## Abstract

Non-destructive measurements of internal morphological structures in plant materials such as seeds are of high interest in agricultural research. The estimation of pericarp thickness is important to understand the grain quality and storage stability of seeds and can play a crucial role in improving crop yield. In this study, we demonstrate the applicability of fiber-based Bessel beam Fourier domain (FD) optical coherence microscopy (OCM) with a nearly constant high lateral resolution maintained at over ~400 µm for direct non-invasive measurement of the pericarp thickness of two different sorghum genotypes. Whereas measurements based on axial profiles need additional knowledge of the pericarp refractive index, en-face views allow for direct distance measurements. We directly determine pericarp thickness from lateral sections with a 3 µm resolution by taking the width of the signal corresponding to the pericarp at the 1/e threshold. These measurements enable differentiation of the two genotypes with 100% accuracy. We find that trading image resolution for acquisition speed and view size reduces the classification accuracy. Average pericarp thicknesses of 74 µm (thick phenotype) and 43 µm (thin phenotype) are obtained from high-resolution lateral sections, and are in good agreement with previously reported measurements of the same genotypes. Extracting the morphological features of plant seeds using Bessel beam FD-OCM is expected to provide valuable information to the food processing industry and plant breeding programs.

## 1. Introduction

Sorghum is the fifth most important cereal crop in the world [1] and an important source of nutrition, especially in Africa and Asia [2,3]. Processing sorghum grain to produce food products usually requires removing the outermost layer, the pericarp of the grain. In sorghum, the pericarp consists of three layers: the epicarp, mesocarp, and endocarp. The mesocarp is the thickest among them and its constituents decide its thickness [4,5]. Pericarp thickness is an important breeding criterium as it is related to different traits, including protection from pests [6], food processing [5,7], storage stability [8], and popping quality [9]. Methods that allow for nondestructive, fast, and cost-effective measurements of pericarp thickness are of interest to assist in the breeding of sorghum varieties with the appropriate kernel characteristics depending on the end-use requirements.

The current methods to measure sorghum pericarp thickness include scraping and removing the pericarp using a scalpel, followed by observation using a magnifying glass [10] and cutting/splitting the kernels in two (or creating slices using a microtome) and assessing the pericarp thickness using light microscopy [11]. It is obvious that these methods are invasive to the kernels and errors can arise easily. A third method uses electron microscopy to scan the sorghum kernels to determine the pericarp thickness, following an assessment of the amount of starch granules in the mesocarp [12]. This method allows for high-precision imaging, but it is also a highly destructive and time-consuming method requiring special sample preparation.

More recently, near-infrared reflectance spectroscopy (NIRS) [11] was implemented to predict the pericarp thickness of sorghum grains. Pericarp thickness depends on the biochemical components present in the pericarp. The contributions to the NIRS spectra of those components were used to estimate pericarp thickness. NIRS was applied to whole grain samples and powdered samples, but the classification between thin and thick pericarp phenotypes performed best for ground samples [11]. It is important to note that this method does not provide a direct pericarp measurement but indirectly calibrates the phenotype in terms of thickness and thinness via modeling based on principal component analysis of the recorded spectra [11]. It is also important to note that these measurements were not performed on individual seeds, but were averaged over many seeds a priori, and the best performance was obtained via destructive testing (ground samples).

Finally, X-ray computed tomography (CT) was also used to phenotypically assess sorghum seeds and their internal structure [13]. X-ray CT uses X-ray images taken from different angles to reconstruct the 3-dimensional structure of objects. While X-ray imaging has the potential to achieve very high spatial resolution, the technology was developed mainly to image large objects, and the detectors are optimized to obtain a moderate resolution. In the reported study, a voxel size (3-dimensional pixel) of roughly 20 µm in every dimension was used, resulting in a spatial resolution limit of roughly 40 µm, which is about the same as the pericarp thickness of the thin pericarp phenotypes reported. Hence, this method will tend to overestimate the pericarp thickness.

A non-invasive method that has been widely applied for the high-resolution non-destructive cross-sectional imaging of biological samples is optical coherence tomography (OCT) [14]. Analogous to ultrasound imaging, OCT imaging obtains depth information by analyzing the transit time of backscattered signals. As the speed of light does not enable the measurement of transit times directly, OCT analyzes the interference with a known reference [15] to obtain a depth profile. Spectral-domain OCT using a broadband light source allows this depth profile (A-scan) to be extracted for any given *x,y*-coordinate in a single shot measurement. Scanning the illumination beam over the *x,y*-coordinates then produces the information to obtain a volumetric image of the sample.

The applicability of OCT to study internal morphological variations in seeds has been demonstrated in several studies, such as measurements of hull thickness in lupin seeds [16], the in situ monitoring of different phases of germination in pea seeds [17], the assessment of morphological modifications induced by viral infections in melon seeds [18], distinguishing between healthy and virus-infected cucumber seeds [19], 3-dimensional imaging of the internal structure of tomato seeds [20], and the identification of fungus-infected tomato seeds [21]. A swept-source OCT setup combined with deep neural network approaches was used for the nondestructive classification of rice grains [22], and a technique dubbed biospeckle OCT [23] was used to study the phenotypical effects of increasing zinc concentrations on lentil seed germination and seedling growth. Pericarp thickness measurements reported by Clements et al. [16] were based on an OCT system providing an estimated lateral resolution of 30 µm and an axial resolution of 24 µm. Axial cross-sections (at the upper surface of the seed) were acquired for the pericarp measurements, requiring an approximation of the pericarp refractive index to determine its physical thickness. Errors in the estimation of the refractive index value of the tissue layers translated linearly to errors in the calculation of the physical layer thickness, and the authors suggest that improvements to the pericarp measurements can be made via direct measurement of the refractive index, adding an additional step to the measurements [24]. Another implementation involving measurements of axial sections at specific locations of the seed coats was performed in pea seeds using swept-source OCT [17] with reported resolutions of 6.3 µm (axial) and 25 µm (lateral). Again, axial cross-sections were used for these measurements; thus, the refractive index of the coat needs to be accounted for to calculate the coat thickness. Both reports [16,17] use an OCT setup with loose focusing of a conventional Gaussian beam profile to avoid strong axial variations in the lateral resolution, and consequently, have a low lateral resolution.

The high-lateral-resolution implementation of OCT is often referred to as optical coherence microscopy (OCM). As a high lateral resolution is typically obtained via imaging through a short focal-length lens or microscope objective, this high resolution is consequently restricted to a short range, referred to as depth of focus (DOF), around the focal plane of the lens or objective. In full-field time-domain OCT [21] this limitation can be resolved through scanning of the focal plane by moving the objective (or sample) and performing only short scans of the reference arm to record interference with backscattered light from the focal plane. In beam-scanning Fourier domain (FD)-OCM, the implementation of Bessel beam (i.e., “non-diffracting” beam) illumination can be used to extend the DOF [25]. While Bessel-beam FD-OCM requires scanning of the laser beam over the sample, no mechanical motion of the reference arm and objective (or sample) is required, and it offers greater sensitivity [26]. In addition to allowing the generation of narrow pencil-like beams with a long depth of focus, another important advantage of Bessel beams is their robustness to tissue scattering [27,28,29]. Low-order Bessel beams from specialty optical fiber allow for a moderate improvement of the DOF, but also have the advantage that they can be focused to a tighter spot than a Gaussian beam from a standard optical fiber [30]. Additionally, for beams guided through the same optical system and of comparable size, the central lobe of a low-order Bessel beam is always smaller than that of a Gaussian beam [30]. As such, replacing a Gaussian beam with a low-order Bessel beam will improve the transverse resolution.

Herein, for the first time, the applicability of a custom-built high-lateral-resolution Bessel-beam FD-OCM system for direct noninvasive measurements of the pericarp thickness of sorghum seeds is demonstrated. By using high-resolution OCM lateral cross-sections instead of axial sections, no additional measurements (or estimations) of the pericarp refractive index are required, making the analysis more accurate and less complex. Measurements with a higher (lateral) resolution enable a lower error in the pericarp measurements, and the observation of finer details in the seeds. To the best of our knowledge, the resolution achieved in our measurements surpasses the resolution used in previous reports of FD-OCT measurements in seeds. This system was used for high-precision measurements of the pericarp thickness in two different sorghum genotypes: BTx2928 [31] and RTx430 [32]. The measurements in this study are consistent with previously observed attributes [13], with the BTx2928 variety having a thick mesocarp (our value: 74 ± 14 µm) and the RTx430 variety having a thin mesocarp (our value: 43 ± 6 µm).

## 2. Materials and Methods

### 2.1. Bessel-like Beam High-Resolution Fourier Domain OCM Setup

**Figure 1:**
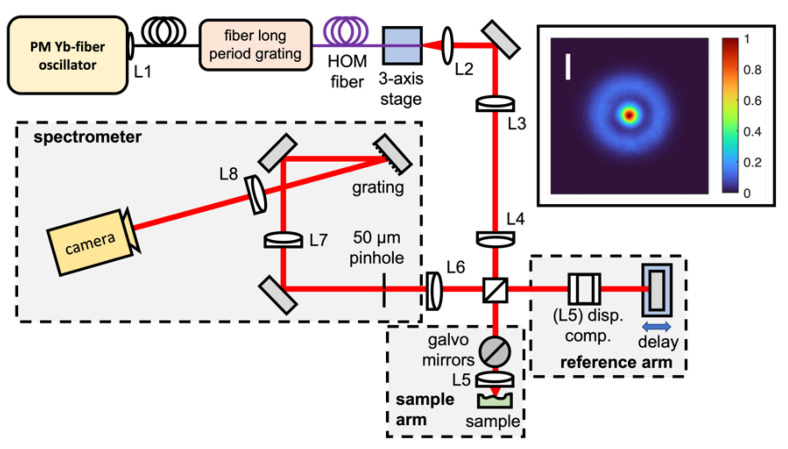
Schematic of the ultrahigh-resolution Fourier domain optical coherence microscopy setup. The inset shows the far-field beam profile of the LP_02_ (Bessel) beam (scale bar 1 mm).

The optical setup (see Figure 1) used to image the sorghum kernels is similar to the setup reported by Sen et al. [30] but was modified to increase the measurement speed more than 1000-fold. Illumination is provided by an all-polarization-maintaining femtosecond Yb-doped fiber oscillator [33] emitting at a central wavelength of 1030 nm. A fiber collimator L1 (Thorlabs F240APC-1064) is used to couple the oscillator output into a polarization-maintaining single-mode fiber (PM-SMF). This PM-SMF is spliced onto the single-mode pigtail of a higher-order-mode (HOM) fiber, which can support four modes, including the Bessel-like LP_02_ mode [33,34,35]. Mode conversion from the fundamental LP_01_ mode to the LP_02_ mode is achieved through long-period grating with a period Λ matching the difference in the propagation constant β of the LP_01_ and LP_02_ modes, which was written into the HOM fiber core using ultraviolet radiation. The output mode of the HOM fiber is shown in the inset of Figure 1.

The output end of the HOM fiber is fixed on a v-groove mounted on a 3-axis translational stage, and the Bessel-like output beam is collimated using an aspheric lens L2 (Thorlabs C240TME-B, f = 8 mm) which is relay-imaged with a 4f-telescope (L3 and L4, Thorlabs AC254-100-B-ML, f = 100 mm) to the Michelson interferometer setup consisting of a 50/50 (Thorlabs BS011) beam-splitter. The sample arm consists of two galvanometric scan mirrors (Thorlabs GVM002), which perform the lateral *x,y*-scans, and a scan lens (L5, LSM02-BB, f ∼ 18 mm). By adjusting the angular scanning range of the galvanometric mirrors, the field of view can be adjusted between < 0.01 mm and 8 mm in both the *x* and *y* directions. A glass block (Thorlabs LSM02DC) is used in the reference arm for dispersion compensation and a flat silver mirror reflects the reference beam. The output of the interferometer is focused on the entrance (Ø 50 µm) pinhole of our custom-made high-resolution spectrometer using lens L6 (Thorlabs LA1027-B, f = 35 mm). The transmitted light is collimated using lens L7 (Thorlabs AC254-200-B-M, f = 200 mm) and diffracted off a 1480 lines/mm gold grating (Horiba Jobin Yvon). The diffracted (spectrally separated) light is focused using lens L8 (Thorlabs AC254-200-B-M) on a high-speed line camera (Teledyne e2v OCTOPLUS). Compared to Sen et al. [27], we used a different camera in the OCT spectrometer and the single-mode fiber output from the laser was not used; only the HOM fiber output was used. The major difference for the spectrometer is that the line camera allows the acquisition of spectra to occur more than 1000 times faster.

The spectral resolution of the spectrometer is 0.2 nm, providing an imaging depth range of ~4 mm. The camera allows for up to 250 kHz A-scan rates at low bit depth (10-bit, maximal 130 kHz @ 12-bit resolution). The lateral resolution of the system is 2.6 µm with a depth of focus of 326 µm, and the axial resolution (in air) of the system is ~20 µm.

### 2.2. Seed Material

**Figure 2:**
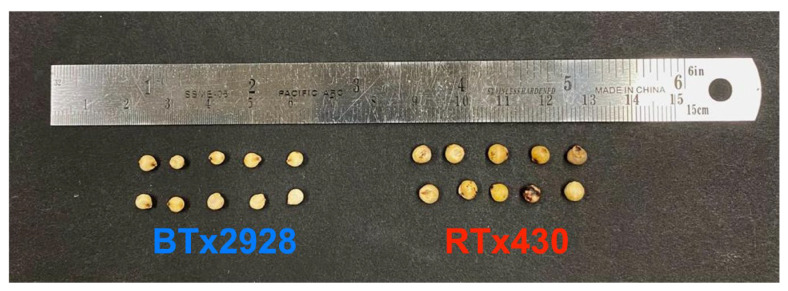
Images of the sorghum grains used in this study: BTx2928 (left) and RTx430 (right).

We compared the pericarp thickness of seeds from two different sorghum genotypes: BTx2928 [31] and RTx430 [32]. Grains for these genotypes were produced in College Station, Texas, USA in 2019 by the Texas A&M AgriLife Research sorghum breeding program. BTx2928 has a thick mesocarp and the RTx430 genotype has a thin mesocarp [13]. Consistently, pericarp characteristics have also been visually described as chalky (thick pericarp) and translucent (thin pericarp). Figure 2 shows the 10 seeds we randomly selected from each genotype for the OCT measurements (20 seeds in total).

### 2.3. Validation Measurements

**Figure 3:**
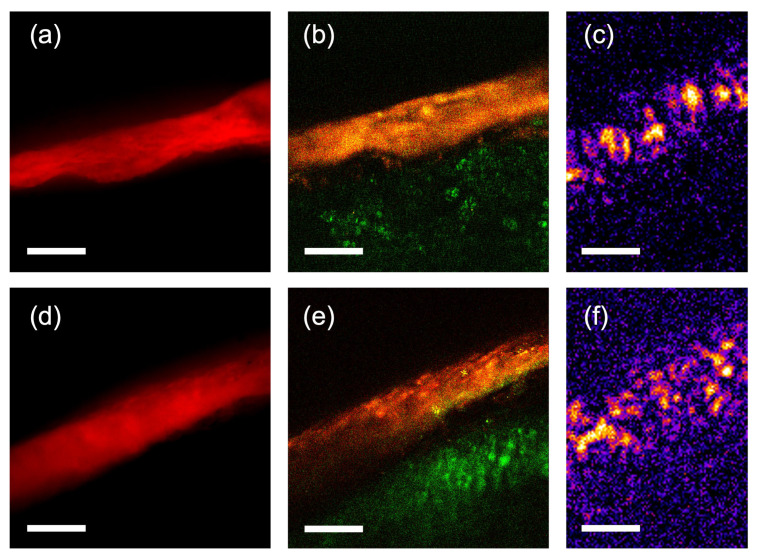
Widefield epifluorescence (**a**,**d**), two-photon microscopy (**b**,**e**), and high-resolution OCT (**c**,**f**) images of an RTx430 seed (**a**–**c**) and a BTx2928 seed (**d**–**f**). The widefield epifluorescence shows red fluorescence from the pericarp of the seed under a 40× objective. The two-photon images show red fluorescence in red, and green fluorescence, as well as second-harmonic radiation generated by the starchy endosperm in green. The red fluorescence in the widefield and multiphoton images allows for clear demarcation of the pericarp. The OCT images display the OCT signal, which originates mainly from the pericarp in a false color representation (FIJI “fire” color scheme). The scale bars in each image represent 50 µm.

A seed from each variety was cut in half after taking the OCT measurements, and the exposed pericarp was directly measured using different methods. Visually (under low magnification in a microscope), the pericarp of RTx430 looks translucent, while the pericarp of BTx2928 looks more opaque. Widefield epifluorescence, 2-photon fluorescence, and second-harmonic generation microscopy images were taken using a home-built multiphoton microscope (based on the modular in vivo multiphoton microscopy system [36]). A sample measurement of the pericarp thickness was determined using two methods (widefield epifluorescence and 2-photon imaging) for both seeds, as shown in Figure 3. As can be observed from these images, local variations in pericarp thickness can be large (the images in panels (a) and (b) show thickness variations between <30 µm and >50 µm within a distance of ~200 µm). Due to these large variations, the fact that our microscope images captured only a limited part of the seed cross-section, the knowledge that our manual cut may or may not be perpendicular to the light propagation in our OCT measurements, and the low precision with which we could control where exactly we cut the seed, it was impossible to directly compare the (widefield and 2-photon) microscopy measurements with the OCT measurements; this is because the exact position of the measurement in the seeds could not be matched. While a large-view measurement of the cut seed (showing the entire seed) would make a direct comparison between the microscope image and OCT measurements less challenging, the coarse resolution associated with such images would hamper the precision. Measurements with a more restricted field of view had a higher resolution (3 µm in our zoomed-in OCT measurements, 0.5 µm in our 2-photon microscopy measurements), and thus, enabled better assessment of the pericarp thickness.

### 2.4. FD-OCM Imaging and Data-Processing

The high-resolution Bessel beam LP_02_ illumination based the OCM setup was used to measure the pericarp thickness of sorghum seeds from the two different genotypes specified above. OCM tomograms of 10 seeds of each genotype were acquired. Tomograms were obtained by acquiring series of A-scans (spectra) with in-house-developed LabVIEW (National Instruments Corp., Austin, USA) software, and subsequently transformed into individual axial profiles using an in-house-developed MATLAB (MathWorks Inc., Natick, USA) script. The processing pipeline was as follows: First a reference spectrum (in our case, the average of all spectra in the acquisition) is subtracted, and an apodization window filter is applied to the result. The resulting filtered baseline-corrected spectra are interpolated onto an equidistant frequency grid, and then Fourier transformed. The absolute value of the first half of the resulting “time domain” signal provides the axial profile (the second half is complementary). For each seed, we acquired 1000 × 1000 A-scans to obtain 1000 × 1000 × 1024 voxel tomograms, which covered a (*x* × *y* × *z*) volume of 5.4 mm × 5.4 mm × 11 mm, enough to visualize the entire upper half of the seeds. We will refer to these tomograms as “entire seed” tomograms. Note that the range in the z-direction is more than the ~4 mm depth range allowed by our spectrometer resolution, as the camera oversampled the spectrum. The time needed for a full tomogram acquisition was ~15 s, which was longer than the 7.7 s that could be achieved at the full speed of the camera (at 12-bit resolution) (or 4 s at a 10-bit resolution) due to limitations of our LabVIEW acquisition software. As a single voxel measures 5.4 µm × 5.4 µm × 11 µm, this resulted in a lateral resolution of ~11 µm and an axial resolution of ~22 µm (in air).

**Figure 4:**
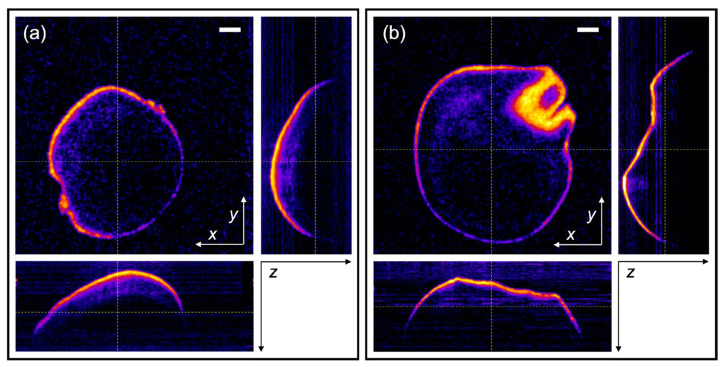
Cross-sections from Bessel-beam FD-OCM tomograms of (**a**) BTx2928 and (**b**) RTx430 seeds. Scale bar: 0.5 mm. The “fire” color-scale from FIJI is used for the false color scheme in the monochrome images (pixel values are scaled according to the logarithm of the OCT signal), helping slightly to enhance small signal details. The position of the *xy*, *xz*, and *yz* cross-sections are indicated with dotted yellow lines in the each of the other sections.

Figure 4 shows the OCM lateral and axial cross-sections of the two different genotypes. To determine the pericarp thickness, the lateral cross-section used was taken at the position where the seeds had the widest perimeter, which could be easily determined for each seed from the tomograms. It can be seen in Figure 4 that especially the hilum region, but also the stylar region, appear to have a different shape than the rest of the seed. As the hilum and stylar regions have more irregular shapes than the rest of the seed, we did not consider them for the pericarp measurements.

Besides “entire seed” tomograms, we also acquired zoomed-in tomograms of 100 × 1000 × 1024 voxels at 10 different positions along the (maximum) perimeter of each seed, each covering a field of view of 0.155 mm × 1.55 mm. A single voxel, in this case, consequently measured 1.55 µm × 1.55 µm × 11 µm. In this case, a total of 10 A-scans were acquired for each pixel, enhancing the measurement contrast. The lateral resolution in these measurements was improved to ~3 µm, which was close to the resolution limit (2.6 µm) of the optical setup. Panels (c) and (f) in Figure 3 show lateral slices of such measurements in both seed varieties.

While not utilized in this work, sampling strategies other than simple raster scanning may allow both an “entire seed” tomogram and high-resolution lateral profiles to be obtained in a single measurement. Real-time processing [37] of the data could be used to identify regions in the volume where a higher lateral sampling density is required, while other regions can be scanned with a much lower sampling density (e.g., around the top of the seed) and where no measurements need to be taken (e.g., outside the seed’s perimeter). With precise positioning of the seeds (e.g., using additional machine vision [38]), it is also possible to automate optimized scan paths [39] over the seed, with a high density of points (and hence, resolution) along the seed’s perimeter, and a lower density of points elsewhere.

### 2.5. Data Analysis and Pericarp Thickness Measurements

**Figure 5:**
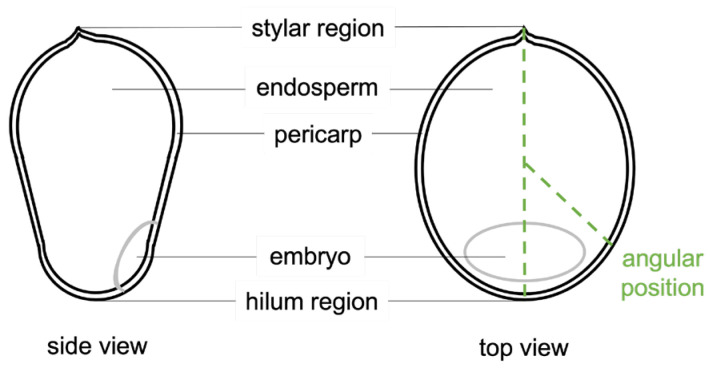
Sketch of side and top cross-sections of a sorghum seed, indicating the pericarp, endosperm, embryo, hilum, and stylar regions. The latter two are on opposing sides of the seed, and we use the midline connecting these two regions to define the positions where we measure pericarp thickness, i.e., at ~30-degree intervals starting at ~30 degrees. The zero- and 180-degree positions are excluded from the measurement as around these positions, more irregularities are found.

From the “entire seed” OCM tomograms, the pericarp thickness was determined by evaluating the OCT signal in lateral cross-sections where the perimeter of the seed showed its furthest extension from the center of the seed. For each seed, 10 positions at different locations (separated from each other by ~30 degrees) were selected to extract the pericarp thickness (schematically indicated in Figure 5). We chose 10 points to allow for analysis and quantification of the thickness variation along the seed’s perimeter, as well as to allow for statistical analysis of the significance of the differences observed. The positions at the tip of the hilum region and the opposing end of the seed were excluded as the shape of the seed in these regions shows more irregularities. The 10 zoomed-in tomograms for each seed were selected to coincide with the 10 positions in the “entire seed” tomograms where the pericarp thickness was measured.

**Figure 6:**
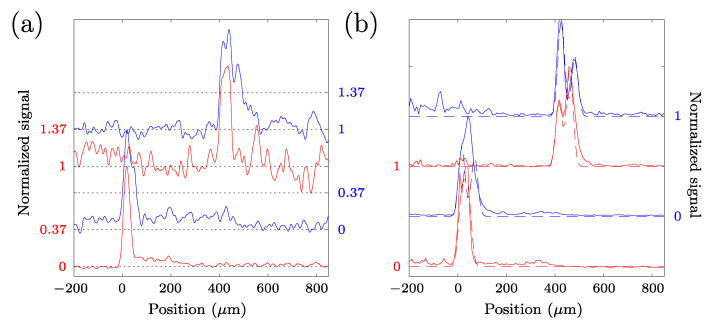
Lateral (**a**) and axial (**b**) profiles from different positions in an RTx430 seed (red traces) and a BTx2928 seed (blue traces). (**a**) The pericarp thickness in the lateral profiles (OCT signal normalized to the peak value in the profile) is determined by the distance between where the signal rises through the 1/e threshold and falls below the 1/e threshold again. The black dotted lines are placed at 0 and 1/e, allowing them to indicate the thickness measurement. The upper traces are vertically offset by 1 from the lower traces. (**b**) The pericarp thickness in the axial traces is determined by the distance between the center of two Gaussian peaks (shown as the dotted traces) matched to the OCT peaks corresponding to the upper and lower boundary of the pericarp, or to the outer edges of the OCT signal peak when the two boundaries cannot be distinguished. Additionally, here, the normalized traces are each vertically offset by 1.

In the lateral cross-section images (en-face views, as shown in Figure 3c,f) where the perimeter of the seed at the selected position was largest, we created linear cross-sectional profiles from the zoomed-in measurements by averaging several lines. Noise on the profiles was reduced by Fourier filtering the profiles, and zero-padding in the Fourier domain allowed for interpolation of the profiles to sub-pixel positions. If the shorter lateral axis of our zoomed-in section was not parallel to the (local) seed edge, but has an angle α, we shifted each line by sin α pixels to align the seed edge in each line. Sub-pixel shifts were made possible by the Fourier interpolation. After summation of all the (shifted) lines, the resulting profile was normalized to the peak maximum. The width of the pericarp was determined by finding the 1/e (~0.367) threshold around the peak of the seed edge. The width of the profile in pixels was converted from the width in pixels to the width in µm by multiplying by the pixel pitch (1.55 µm) and cos α, to correct for the angle between the short axis of the measured cross-section and the seed edge. Figure 6a shows examples of the lateral profiles obtained for each variety. It is important to note here that while in other research, the hull thickness could be determined by the distance of two distinct, local maxima in the OCT signal, in our case, the more uniform scattering throughout the pericarp region at most times caused a broader, uniform peak spanning the pericarp region.

From the axial cross-sections near the location in the seed closest to the focusing lens of our OCT system, we also analyzed the profile to retrieve the thickness of the pericarp of the seeds. For a couple of seeds, this measurement failed because the top surface was too close to the zero-delay between the sample and the reference arm. From the measurements where this was not the case, we extracted the thickness of the pericarp layer by assuming a pericarp refractive index of 1.33 and matching the sum of two Gaussian peaks with a 1/e width of 18.8 µm (corresponding to the axial resolution of our system in a medium with a refractive index of 1.33), variable height, and a variable center position to the axial profiles. The values obtained using this method were consistently smaller than the average values obtained from the lateral cross-sections (except for one instance where it coincided with the average value). In Figure 6b, we show examples of the axial profiles obtained from the different genotypes.

### 2.6. Statistics

We calculated the average pericarp thickness evaluated using both the coarse- and high-resolution measurements, both for each seed and for all the seeds of each genotype. We also calculated standard deviations and performed Student’s *t*-tests on the data sets to confirm the significance of differences between genotypes, using MATLAB’s internal functions (std and ttest2, respectively).

## 3. Results

**Figure 7:**
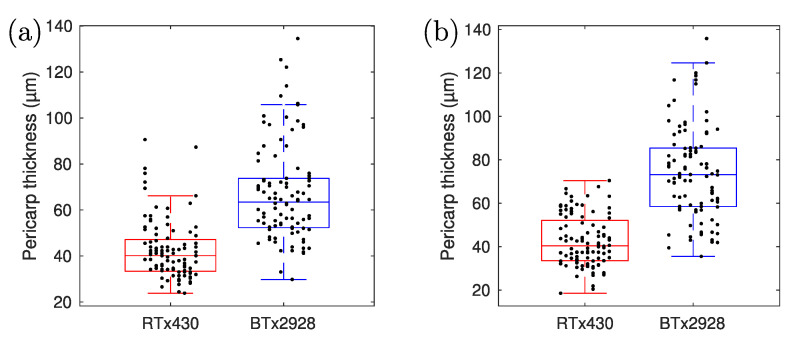
Box-and-whisker plots combining all pericarp thickness measurements with (**a**) 11 µm and (**b**) 3 µm lateral resolutions. The *p*-value obtained from Student’s *t*-test for the large-field-of-view measurements is 3.2 × 10^−19^ (**a**), and for the zoomed-in measurements (**b**) the *p*-value is 1.9 × 10^−29^.

From the “entire seed” tomograms (with 11 µm lateral resolution, see Figure 4), the average value of the measured pericarp thickness of the BTx2928 seeds is 67 µm, with a standard deviation of 10 µm. The average measured pericarp thickness of the RTx430 seeds is 42 µm, with a standard deviation of 9 µm (Figure 7a). Student’s *t*-test (*p*-value: 3.2 × 10^−19^) shows a clear differentiation in pericarp thickness between these two genotypes. From these tomograms, we also evaluated the axial cross-section at the “upper” region of the seed, yielding an average thickness of 40 ± 7 µm for the BTx2928 seeds and 29 ± 7 µm for the RTx430 seeds. Four representative axial profiles are shown in Figure 6b.

From the zoomed-in tomograms (with 3 µm lateral resolution), the average measured pericarp thickness of the BTx2928 seeds is 74 µm (standard deviation of 14 µm) and the average measured pericarp thickness of the RTx430 seeds is 43 µm (standard deviation of 6 µm). These results are shown in Figure 7b. Representative views for each genotype are shown in Figure 3c,f, and four representative lateral profiles are shown in Figure 6a. Again, Student’s *t*-test on the data (*p*-value: 1.9 × 10^−29^) clearly shows the statistical differences between genotypes.

**Figure 8:**
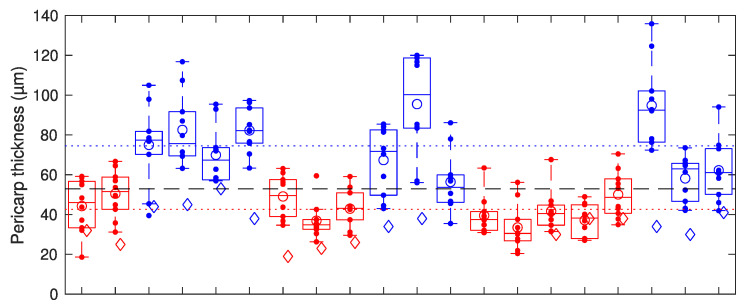
Plot showing the variation in pericarp thickness within each seed measured using the zoomed-in tomograms. Red points are for the RTx430 seeds, blue points are for the BTx2928 seeds, and points that are vertically aligned are for the same seed. Horizontally, the seeds are displayed according to the order in which they were measured. Open circles show the average for each individual seed. Boxes and whiskers show the median and quartiles for each seed, where 2 out of 200 measurements could be considered outliers. The red and blue dotted lines show the average pericarp thickness from the zoomed-in lateral measurements for the RTx430 and BTx2928 seeds, respectively. The black dashed line shows the threshold value that can be used to classify the seeds (see text for classification rules). For comparison, the open (red and blue) diamonds show the pericarp thickness values extracted from axial measurements.

The 10 high-resolution pericarp thickness measurements for each seed can be seen in Figure 8. From the zoomed-in lateral measurements, seeds can be classified based on their average pericarp thickness. The average pericarp thickness for all the RTx430 seeds is below 53 µm, while all the BTx2928 seeds have a pericarp thickness above 53 µm.

**Figure 9:**
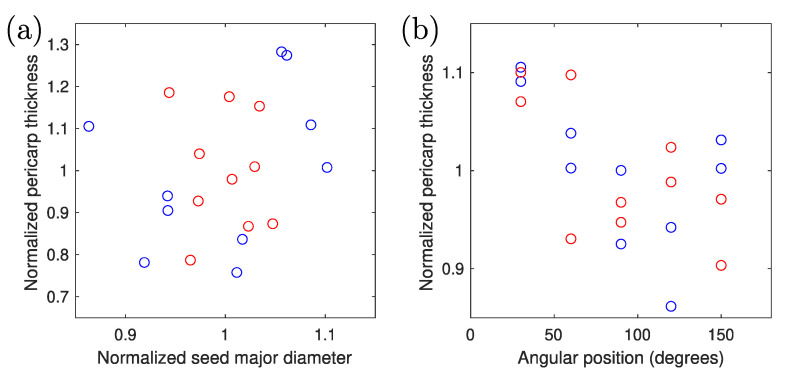
Pericarp thickness compared to seed size (**a**) and position (**b**) along the seed perimeter. Red symbols are derived from RTx430 seed data and blue symbols from BTx2928 data.

As noticeable differences in size between seeds of the same genotype can be observed, and we also tested whether the average pericarp thickness correlates with the seed’s major diameter for each genotype. With a Pearson’s correlation coefficient of 0.24, no such correlation is established (Figure 9a). Moreover, from our measurements, with a Pearson’s correlation coefficient of −0.6, no significant trend in pericarp thickness vs. distance from the hilum region is established (Figure 9b). Although a slight decrease in average thickness is visible, it also has to be noted that the error bar for each of the data points (as given by the standard deviation of the 10 measurements) is much larger than the apparent decrease.

## 4. Discussion

Our Bessel-like beam OCM clearly shows that kernels from BTx2928 have a thicker pericarp than those from RTx430. These observations are consistent with visual observations (BTx2928 has a chalky pericarp and RTx430 has a translucent pericarp), and with the CT-based observations [13] of a thin mesocarp for RTx430 and a thick mesocarp for BTx2928. The CT-analysis reported average mesocarp volumes for each variety, which made straightforward conversion between the two quantities a bit more challenging, as the volume can increase with seed size even when the thickness decreases. To address this, one can assume an ellipsoidal shell model (see Appendix A) to facilitate the conversion of the results of our measurements to a pericarp volume (BTx2928: 1.71 mm^3^; RTx430: 1.61 mm^3^), which shows that our results are consistent with the pericarp volume measurements presented in [13]. The reason our measurement yields a significantly smaller pericarp volume than the value obtained using CT [13] for RTx430 could be that the coarse spatial resolution of the CT measurement (being almost equal to the pericarp thickness) leads to larger uncertainty and overestimation in the measurement of the volume of a thin shell with CT.

While in many OCT measurements from seeds, clear peaks in axial cross-sections can be observed and interpreted as demarcations of the seed coat, hull, or pericarp regions, in our measurements, typically, only one broad peak at the seed perimeter is observed. This has several reasons, the first of which is the relatively low axial resolution of our setup (~20 µm inside the seeds), in connection with the small thickness of the different layers in the pericarp. Thus, as individual layers are spaced by less than the axial resolution, the OCT peaks from each interface overlap with the neighboring peaks, resulting in a broader peak, as can be observed in Figure 6b. Second, the lateral cross-sections from our retrieved tomograms also only reveal a broad peak at the pericarp layer, indicating a high degree of scattering from within the cells in the pericarp layer, and a much lower degree of scattering from the endosperm region. Inspection of high-resolution multiphoton microscopy images of the exposed (after cutting the seed with a knife) cross-section also reveals strong scattering of the pericarp region and a fine structure of the pericarp cells beyond the resolution of our OCT measurements. Moreover, the local variation in the pericarp thickness observed in these measurements is supportive of the large variance in the thickness measurements along the seed perimeter using OCT; this is due to true variations in the thickness rather than measurement uncertainty. Figure 3, Figure 7 and Figure 8 reveal that local variations in pericarp thickness within a seed can be larger than the variations in average pericarp thickness between seeds of the same variety. This observation is in line with measurements performed using scanning electron microscopy where significant pericarp thickness variations within an individual kernel are reported [12] in sorghum varieties.

The chosen method for the analysis of the axial and lateral profiles in the OCT measurements can influence the exact measured values. The method we selected to analyze the lateral profile data disregards that the thickness can be slightly overestimated as, in fact, the true profile is convoluted with the instrument response (resolution). With our measurements, this instrument response function becomes wider as a function of distance from the geometric focus of the OCT scan lens [30], but much less so than for an OCM system using Gaussian illumination. With most of our measured lateral profiles, the lateral instrument response width only merits an insignificant correction to the measured width; hence, we have not included this correction.

Other possible models to retrieve the pericarp thickness include measuring the centers of two Gaussian signals with a fixed width fitted to the outer edges of the OCT signal corresponding to the pericarp region, or fitting a Gaussian peak with variable width to this signal, where the 1/e full width corresponds to the pericarp thickness. The method of choice for our analysis (defining the thickness by thresholding the normalized data), has a lower tendency to overestimate the thickness than using the width of a single Gaussian function fitted to the OCT signal, which, on average, yields a thickness 5 µm larger for both varieties.

When it comes to axial profiles, fitting Gaussian peaks with a fixed width (corresponding to the axial resolution or instrument response—roughly ~20 µm in air, with our system) to the leading and trailing edge of the axial profiles is the method of choice to determine layer thickness. A higher axial resolution can be achieved when using a light source with a greater spectral bandwidth and replacing the grating in the spectrometer with one that has lower groove density to accordingly increase the spectral range covered. In the analysis of axial profiles, multiple scattering points axially separated by less than the axial resolution will cause distortion of the axial profile, which, in turn, can lead to errors in the extracted thickness; depending on where the scattering happens, this can lead to under- or overestimation of the pericarp thickness. As the results from our axial measurements are consistently smaller than those from our lateral measurements, we believe that in our case, the thickness is underestimated due to multiple scattering layers within the pericarp. Additionally, poor knowledge of the refractive index of the layer to be measured causes additional uncertainty in the extracted values. As our choice to use the refractive index value (*n* = 1.33) of water for this estimate is relatively low (this value could be considered the lower bound for the tissue estimate), this leads to overestimation of the pericarp thickness, as the speed of light in a medium inversely scales with the refractive index. While the axial profiles yield significantly different thickness values for the pericarp layer than the lateral profiles (29 ± 7 µm axial and 43 ± 6 µm lateral for the RTx430 seeds; 40 ± 7 µm axial and 74 ± 14 µm lateral for the BTx2928 seeds), the values are consistent with the observation that the BTx2928 seeds have a thicker pericarp than the RTx430 seeds. 

Optical coherence microscopy allows for direct, non-contact determination of the pericarp thickness of sorghum seeds. As we acquired measurements from 10 different positions along the seed perimeter, this required about 2.5 min of measurement time per seed. From coarser measurements that cover the entire seed in only 15 s, slightly different values of 67 ± 10 µm for the BTx2928 variety and 49 ± 9 µm for the RTx430 variety are obtained. Even shorter measurement times, e.g., 1 s or less per seed, can be achieved with appropriate software optimization and optimized sampling strategies. In fact, our camera supports a roughly 4-times-higher A-scan rate than what we achieve with our current data-acquisition software. For this work, the data processing was performed using in-house-developed MATLAB scripts, and the resulting images were analyzed using FIJI and MATLAB. Optimized software dedicated to OCT will allow for much faster and automated data analysis. Real-time data processing of OCT measurements and automated, real-time extraction of features of interest from the resulting imagery can be readily achieved and are already available commercially for other applications. Real-time processing of the measurements [37] (optionally combined with additional machine vision [38]) can also be utilized to implement optimized scan trajectories [39] that allow visualization of the entire seeds (at a low resolution), while at the same time, acquiring high-resolution data at the seed’s perimeter. The galvanometric mirrors used in our work can be readily programmed to scan custom trajectories that realize the above strategy.

## 5. Conclusions

In this work, we demonstrate, for the first time, the applicability of a high-lateral-resolution Bessel-beam FD-OCM system for direct noninvasive measurements of (physical) pericarp thickness in sorghum seeds. As these measurements can be performed in lateral cross-sections (en-face views), no additional measurements or estimation of the pericarp refractive index is required. This makes the analysis more accurate and less complex than measurements of seeds using OCT in other reports where axial cross-sections were analyzed. By taking kernel cross-sections perpendicular to the light propagation direction, we also take advantage of the high lateral resolution of the system. The higher lateral resolution reduces overestimation of the pericarp thickness measurement. It also allows for higher precision in the measurements of the pericarp at specific points as compared to other presented FD-OCT studies where axial scans are employed. In addition, the long depth of focus of the system maintains a high transverse resolution over a substantial depth range (400 µm), ensuring that the measurement results are not influenced by slight vertical offsets between seeds. Adjusting the transverse scanning parameters increases the covered volume and imaging speed of the OCT tomograms at the cost of transverse resolution. In our work, measurements were taken at two different lateral resolutions (11 µm and 3 µm), which both gave consistent results. Using OCM, the pericarp thickness in sorghum was found to be variable between seeds of the same genotype, as well as within a seed. Thus, multiple seeds and positions along the seed’s perimeter must be measured to reduce the adverse effects of local variation in pericarp thickness. Using the approach presented herein, statistical differences were detected in pericarp thickness between two sorghum genotypes, which allowed for 100% seed classification accuracy. OCM can be applied in breeding programs and plant physiology studies to obtain high-resolution 3-dimensional images of seeds and potentially reveal new traits.

## Data Availability

Data underlying the results presented in this paper are not publicly available at this time, but may be obtained from the authors upon reasonable request.

## Appendix A

In order to compare the reported mesocarp volumes [13] to the pericarp thickness measured in our work, we estimated the pericarp volume of the seeds we measured by employing an ellipsoidal model, i.e., calculating an ellipsoidal shell with dimensions *a*, *b*, and *c* along the *x*, *y*, and *z* directions, and a thickness of *d*. The dimensions of the seed were extracted from our OCT measurements, with *a*—half the length of the seed along the hilum–stylar line, *b*—the half-width perpendicular to this line (both in the lateral cross-section where the seed perimeter is largest), and *c*—the distance from the slice where the perimeter is largest to the slice that just reveals the top of the seed. With this, we calculated the surface area of the seed, and the pericarp volume V was estimated by multiplying this area by the pericarp thickness *d:*

(A1) $$V\approx 4\;d\pi \sqrt[{p}]{\frac{a^{p}b^{p}+a^{p}c^{p}+b^{p}c^{p}}{3}}$$

where setting the parameter *p* = 1.6075 allows for a relative error <1.1% for the approximation.
